# Gender stereotypes regarding power and niceness in Japanese children

**DOI:** 10.1098/rsos.230863

**Published:** 2024-07-24

**Authors:** Xianwei Meng, Mako Okanda, Yasuhiro Kanakogi, Moe Uragami, Hiroki Yamamoto, Yusuke Moriguchi

**Affiliations:** ^1^ Graduate School of Informatics, Nagoya University, Nagoya, Aichi, Japan; ^2^ Graduate School of Psychology, Otemon Gakuin University, Ibaraki, Osaka, Japan; ^3^ Graduate School of Human Sciences, Osaka University, Suita, Osaka, Japan; ^4^ Department of Human Sciences, Sugiyama Jogakuen University, Nissin, Aichi, Japan; ^5^ Department of Psychological and Brain Sciences, Indiana University, Bloomington, IN, USA; ^6^ Japan Society for the Promotion of Science, Chiyoda-ku, Tokyo, Japan; ^7^ Graduate School of Letters, Kyoto University, Kyoto, Kyoto, Japan

**Keywords:** gender stereotype, gender inequality, social power, belief, social development

## Abstract

Belief in gendered social power imbalance (i.e. males are more powerful than females) leads to undesirable gender disparities, but little is known about the developmental origins of this belief, especially in Eastern cultures. We investigated the development onset of this belief by focusing on 4–7-year-old Japanese children while considering another belief (females are nicer than males) for comparison. In the dyadic context tasks, children saw pairs of animated characters depicting powerful–powerless or kind–unkind postures and judged the characters' gender (boy or girl). Results suggested both ‘nice = female’ and ‘powerful = female’ gender stereotypes in children. In the collective context tasks, children were presented with stories in occupational contexts, including multiple unspecified people and verbal cues, describing more explicitly the powerful and nice traits of the protagonists. The results replicated the ‘nice = female’ gender stereotype. Moreover, early ‘powerful = male’ gender stereotypes were seen in 6-year-old boys but not among girls in general. These findings demonstrate that Japanese children's beliefs regarding gender differences in power vary depending on the context in which male–female interactions are presented. Additionally, the study reveals that signs of the ‘powerful = male’ social power gender stereotype emerge around the age of 6.

## 1. Introduction

### 1.1. Gendered social power imbalance and people's beliefs

Imbalanced social power (e.g. greater influence, greater decision-making) between men and women has been observed widely throughout human history: men generally benefit from higher social status, controlling more of the public sphere [1–4]. In the last few decades, although there has been a stronger commitment to promoting gender equality, gender disparity in social status persists strongly, even in many developed countries [5]. Only 58 women have ever served in the United States Senate (out of approximately 2000 senators; [6]). Moreover, in 2021, only 37 Fortune 500 CEOs are women (the highest number yet recorded [7]). In the academic community, climbing the hierarchy remains challenging for women: only 36% of full professors and 30% of college presidents are women [8–10]. In Japan, women currently hold only 23% of the seats in the House of Councilors (the upper house of the National Diet of Japan). Similar to the USA, in Japan, the number of female company presidents has reached its highest level in 2021, though they remain a small minority at 8.1% [11]. Regarding leading academic positions in Japan, a mere 20% and 12% of full professors and college presidents are women, which is lower than that in the USA [12].

What triggers the gender hierarchy in social power? One crucial factor might be people's beliefs about how women and men differ and the behavioural norms that shape different gender roles [13,14]. Beliefs about gender differences have been studied as ‘gender stereotypes’, that is, a constellation of traits and roles generally attributed to either men or women [15]. Past studies have shown that across various cultures, people hold biased beliefs about gender differences; for example, men are more intelligent, competent and ambitious, while women are more supportive, caring, warm and emotional. Gender stereotypes have been identified as a factor influencing people's inclination to self-select into specific activities, resulting in undesirable gender disparities. For instance, the stereotype equating ‘brilliance’ with males harms the empowerment of women and girls in science, technology, engineering and mathematics [13,14,16–19].

In terms of gender imbalance in social power, certain gender stereotypes may limit the interest of women and girls in assuming leadership roles and hinder their performance in high-ranking positions. For instance, since competence and intelligence are widely recognized as essential traits for leadership [20], the stereotype linking ‘competent and/or intelligent’ with males (e.g. [21]) may diminish women's and girls’ motivation to compete against males due to feelings of inferiority. Furthermore, according to the ‘goal congruity model', individuals' motivations for pursuing leadership positions are influenced by their endorsement of the goals associated with those roles [22]. Research has indicated that women show increased interest in political roles when they are framed as serving the community, aligning with goals consistent with gender stereotypes and values associated with females [22–25]. Given that individuals' endorsement of goals can be significantly influenced by the gender stereotypes they hold [26], the goal congruity model supports the idea that certain gender stereotypes contribute to gender imbalances in social power.

The current study focused on another significant gender stereotype: the ‘powerful = male’ stereotype, which suggests that people attribute more social power to men than women [15,27–30]. This stereotype may directly constrain the interest of women and girls in assuming leadership roles, thus exacerbating gender inequality in social power. People who hold the ‘powerful = male’ gender stereotype are more likely to follow norms such as ‘women are proscribed from exhibiting high-status signs of dominance, while men are prohibited from showing low-status signs of weakness' [31]. The impact of holding the ‘power = male’ gender stereotype on gender imbalance in social power may emerge during early development. From the preschool period, children spend longer engaging in social interaction with peers outside their families [32]. Studies have shown that these interactions are generally formed under hierarchical structures such as leader–follower peer relationships [33–35] and often involve exclusion [36]. Therefore, if children internalize and act upon the ‘powerful = male’ gender stereotype, many girls are likely to avoid leadership positions, even in peer interactions. This avoidance may subsequently impact their future choices of social roles in various contexts, potentially diminishing their desire and perceived capability to pursue leadership roles in the workplace. Thus, investigating whether, when and to what extent the stereotype of social power emerges in early childhood contributes not only to drawing a fuller picture of how the ‘powerful = male’ gender stereotype develops but also to developing practical interventions that may reduce the biased belief and avoid its by-product of prejudice and discrimination in childhood as well as the possible biased choices in future.

### 1.2. Development of in-group favouritism and ‘powerful = male’ stereotype

Behavioural studies have shown that infants exhibit in-group bias and discrimination effects (e.g. 5–6-month-old infants prefer to look at a person who previously spoke their native language [37,38]). With development, children's in-group favouritism extends to gender, race and more arbitrary categories (e.g. having physical commonalities such as dressing in the same T-shirts) [39]. ‘In-group love’ makes children favour in-group members [40] over out-group members in various situations, such as when deciding whom to give valuable resources [41], to play with [42] and to trust [39]. Moreover, in-group favouritism is often observed to be stronger in girls than boys [43].

As children develop, their social judgements not only reflect in-group favouritism but also encompass their beliefs about how their group is perceived by others (e.g. whether the in-group is viewed as better/worse than the out-group) [44]. For instance, American children attribute their own gender as more brilliance than the opposite gender at 5 years of age, whereas by 6 years of age, the in-group favouritism in girls starts to decline to chance, possibly because children from this age hold a ‘brilliance = males’ stereotype [45]. Meanwhile, whether children thought their gender was ‘nicer’ than the opposite gender showed a reversed pattern; the in-group favouritism in girls persisted across different ages, while for boys, it diminished with development [16,45].

The impact of the interaction between in-group favouritism and gender stereotypes on social judgements of power has been observed in past investigations. Mandalaywala *et al*. [46, experimant 1] investigated how children's own group memberships influence their judgements of status beliefs. In the experiment, children aged 3.5–6.9 years from diverse racial-ethnic backgrounds in Manhattan were tasked with positioning characters on a rope. In this task, a higher position symbolized higher status, granting access to resources and decision-making power. The study found that boys were expected to hold higher status: male participants positioned the boy character higher than the girl characters, while female participants did not prioritize the girl characters over the boys. Interestingly, gender-related status beliefs did not correlate with gender-related social preferences; instead, children tended to prefer members of their own gender regardless of their perceived status. Similarly, Santhanagopalan *et al*. [47] demonstrated that when 5–10-year-old Indian children were asked to draw a ‘leader', none of the boys depicted a girl, while approximately half of the girls drew a female leader. Additionally, Charafeddine *et al*. [48, experimant 3] examined 4–5-year-old French and Lebanese children, asking them to determine which puppet, a girl or a boy, held a powerful position after listening to a sequence of dialogues between the puppets. The study found that while a majority of boys associated the boy character with dominance, girls' responses were at chance levels.

Charafeddine *et al*. [48, experimant 1] tested the ‘powerful = male’ belief using stimuli of child characters with limited gender-related information [48]. In the experiment, participants were presented with a drawing depicting an interaction between two non-gendered child characters, with one character displaying a dominant body posture and the other a subordinate posture. The findings revealed that from the age of 4, regardless of the participants' gender, children from France, Lebanon and Norway were more inclined to link the dominant character with male. This suggests that pre-schoolers across different cultures tend to attribute more power to males in mixed-gender dyadic interactions.

These findings suggest that, by the age of 4, both in-group favouritism and the ‘powerful = male’ gender stereotype are evident, particularly among girl participants. This results in different gender-based judgements about social power between boys and girls. Boys consistently attribute power to male characters, reflecting their in-group favouritism and/or beliefs in the ‘powerful = male’ stereotype. In contrast, girls' judgements based on the ‘powerful = male’ stereotype and their in-group favouritism bias are obstructed by each other, often resulting in overall judgements being at a chance level. Furthermore, limiting gender-related information may reduce girls' own-gender bias, making their judgements based on the ‘power = male’ stereotype more apparent.

### 1.3. Limitations of understanding in Japanese culture

However, children's view of ‘powerful’ may differ between Japanese and Western cultures. Charafeddine *et al*. [49] found an early-emerging cross-cultural difference in children valuing dominance. In the study, 3- and 5-year-old pre-schoolers in France and Japan were asked to identify with either a dominant or a subordinate (the stimuli were identical to that of Charafeddine *et al*. [48]). French pre-schoolers identified themselves with the dominant, but Japanese pre-schoolers' judgement was at chance. Further investigations revealed that Japanese pre-schoolers were likelier to believe a subordinate than a dominant individual, compared with chance and previous findings among French pre-schoolers [49]. This suggests that pre-schoolers from Japan value dominance less and are more likely to trust a subordinate individual than a dominant individual compared with their counterparts from a Western culture (i.e. France). The findings were consistent with the notions that Eastern cultures, such as Japanese and Chinese cultures, negatively evaluate the selfish behaviour of the dominant and relatively value the prestigious individuals who modestly yield to subordinates [50].

These suggest that Japan would be a valuable culture to provide insights on the developmental trajectory of children's gender stereotype of power. Specifically, as Japanese children do not value dominance over subordinate characters, their social judgements towards the characters would not be influenced by in-group favouritism [49,50]. This may enable us to investigate children's ‘powerful = male’ gender stereotype in a more neutral context compared with studies in Western countries. Special benefits would arise if boys still showed ‘powerful = male’ judgements, as it would allow us to conclude that this reflects ‘powerful = male’ gender-stereotyped beliefs rather than in-group favouritism. For girls, if they hold ‘powerful = male’ gender stereotypes similar to those of participants in past studies in Western cultures, then the ‘powerful = male’ judgements would be very apparent because biased judgements would not be moderated by in-group favouritism. Together, investigations on children in Japanese culture would significantly contribute to understanding of the development of the ‘powerful = male’ stereotype (as well as the universality versus cultural specificity regarding the development). As Charafeddine *et al*. [49] did not ask children to associate the dominant and subordinate characters with gender, whether the ‘powerful = male’ stereotypes observed in Western cultures can also be found in Japanese children is still an open question.

Together, the first limitation of this topic is that no investigation has been conducted to test whether the ‘powerful = male’ stereotype observed in Western cultures (i.e. children link boy and girl characters to powerful and subordinate in dyadic interactions, respectively) could be found in Japanese children [48].

The second limitation is that it was unclear how Japanese children associate gender with power in the adults' occupational contexts. For investigation in the USA, Reyes-Jaquez & Koenig [51] tested whether children use other individuals’ gender to predict who is in charge and how power is gained in occupational contexts. The study found that 6–10-year-old American boys (but not girls) associated power with adult workers of their gender and did so regardless of whether power had been obtained meritoriously (Experiment 1). These findings suggest that in Western cultures, by the elementary school years, children's associations between gender and power in occupational contexts vary depending on the children's gender. Furthermore, the gender-related judgements about power in occupational contexts seem to align with research documenting the development of the ‘powerful = male’ gender stereotype in dyadic contexts, as mentioned in previous studies [46–48].

In 2023, the World Economic Forum reported that the gender gap in Japan was ranked as low as 125th out of 146 countries (with the USA ranked 43rd), and the Political Empowerment ranking was especially low at 138th (with the USA ranked 63rd) [52]. This suggests that Japanese children are often exposed to the asymmetric power balance between males and females regarding occupational contexts, such as the fact that the most powerful politicians who appear on television are males. As a result, Japanese children are expected to hold a ‘powerful = male’ stereotype regarding occupational contexts, which may be more apparent than suggested by investigations in the USA [51]. Examining this stereotype is important because occupational contexts provide an unambiguous and legitimate context of hierarchy (i.e. an institution with an organizational structure that includes multiple people serving as superiors and subordinates), and social power generally links to status in such hierarchical systems rather than dyadic interactions [53,54]. Furthermore, although children associate children's gender with social power [48], children's responses in those settings may have mainly reflected participants' reasoning about peer relationships rather than the societal power dynamics children witness with adults [55]. Therefore, children's gender stereotypes in collective and dyadic contexts might reflect different evaluation processes. However, whether Japanese children associate social power with males in collective contexts has not been investigated.

### 1.4. Development of ‘nice = female’ stereotype

Fiske *et al*. [16] contended that in interpersonal and intergroup settings, individuals exhibit a primary interest in the characteristics of others, which aligns with the two fundamental dimensions of stereotypes—warmth and competence [16]. Warmth is associated with good intent, while competence is linked to high capability. Correspondingly, Bakan [56] proposed two modalities of human existence closely tied to the warmth–competence distinction: communion (reflecting connection with others) and agency (pertaining to self-promotion [57,58]). Many studies have demonstrated that females value communal goals more than males, exemplified by their endorsement of helping others as a life goal [22–24]. In the context of gender stereotypes, women are stereotypically perceived as warmer (e.g. kind and nurturing) than men [59].

This belief has been observed in the preschool period within Japanese cultural contexts [60] (also supported by evidence in American culture [45]). In Okanda *et al*.'s study [60], Japanese children responded to gender stereotype questions by choosing stick-figure stimuli representing males and females and chose the female stimuli more frequently when they were asked to indicate which stick-figure was ‘really, really nice’ [45].

The methodology and findings have the potential to contribute significantly to investigations into the development of the ‘powerful = male’ gender stereotype in Japanese culture. Specifically, the niceness task may enable the comparison of children's attribution of social power and other traits (e.g. niceness). If children's developmental trajectories of gender stereotypes vary by traits, then it would suggest that the ‘powerful = male’ belief belongs to a specific domain of gender stereotypes that may have a specific developmental onset; this form of comparison has been applied in past studies exploring children's gender stereotypes [45,60]. Furthermore, it is valuable to conceptually replicate Okanda *et al*. [60] and determine whether the current study shows the same pattern in children's gender stereotypes about niceness. If it does, we can conclude that the current experimental procedure and participant sample did not deviate from past investigations.

### 1.5. The current study

The current study assessed whether and from what age Japanese children develop gender-based stereotypes relating to social power and niceness regarding the dyadic male–female interaction context [48] and the occupational collective context, respectively.

Regarding the social power stereotypes, the dyadic context task used the stimuli of Charafeddine *et al*.'s previous investigations [48,49,61]. Children judged which of two characters—one expressing a dominant body posture and the other expressing a subordinate body posture—represented either a boy or a girl. The collective context task was based on the tasks in the study of Bian *et al*. [45] and Okanda *et al*. [60], who tested children's gender stereotypes about intellectual ability. Children were shown stories depicting a powerful person in a group context that included multiple unspecified people. Then, the children judged the gender of the target persons.

In investigating niceness stereotypes, we aimed to conceptually replicate the findings of Okanda *et al*. [60], who demonstrated the ‘nice = female’ gender stereotype in Japanese children from 4 years of age. In the current study, two niceness tasks were conducted; one followed the dyadic power task, and another followed the collective power task. In the former nice task, children were given a drawing of two characters interacting, with one acting kindly and the other unkindly. Then, the children were told that the picture depicted a girl and a boy and were asked to indicate which was which. In the latter nice task, children were shown stories depicting a nice person in a group context that included multiple unspecified people. Then, the children judged the gender of the target persons. The power tasks were always conducted before the nice tasks. This was not only because our main interest was children's judgements on the power tasks but also because the past study using a similar methodology did not find the influence of task (context) order on children's judgements [60].

Okanda *et al*. [60] have suggested that stick-figure stimuli are an appropriate cue for testing Japanese children's gender stereotypes. Specifically, children correctly identify the gender of stick-figure stimuli and can respond to gender stereotype questions (e.g. answer which of the gendered stick figures is ‘really, really nice’). Interestingly, the study suggested that stick-figure stimuli seem more efficient as cues for eliciting children's stereotypes than photos of males and females. Specifically, children showed clearer ‘brilliance = male’ stereotypical responses when presented with stick-figure stimuli than photos of men and women. The authors explained this finding by considering that compared with real photos, stick-figure stimuli may convey more abstract information about gender with minimal noise (e.g. visual information related to clothes and hairstyles), helping children answer questions by focusing on the concepts of gender [60].

The current study included 4–7-year-old Japanese children as participants. The age range was determined for the following reasons: Okanda *et al*. [60] showed that Japanese children at this developmental stage can respond to gender stereotype questions by choosing stick-figure stimuli representing females and males. Therefore, this age range is considered appropriate for the methodologies used in the current study. Moreover, Charafeddine *et al*. [48] showed that children from 4 years of age from France, Lebanon and Norway associated the dominant posture more frequently with boys than girls. Therefore, the age range of the current sample could provide developmental patterns of the ‘powerful = male’ gender stereotype comparable to other cultures.

We hypothesized that children would be more likely to identify the dominant characters as males in the *power tasks* and that they would be more likely to identify the nice characters as females in the *nice tasks* [45,60,62]. Further, theoretical and empirical evidence has suggested that children learn about gender differences from their environment and the accumulation of experience, which largely depend on age [45,60,63–68]. Therefore, we expected an age effect on children's judgements in which older children would be more likely to show gender stereotypes [63–66]. The above hypotheses would be reflected by the results in which children's judgements will be influenced by factors related to task and age.

Specifically, in both the dyadic and collective contexts, because the dependent measure of the tasks was whether children judged the character as their own-gender, we expected that boys and girls would show reversed judgement (i.e. if boys hold the ‘powerful = male’ gender stereotype, then the outcome of their own-gender judgement would be 1 = yes; if girls hold the stereotype, their own-gender judgement would be 0 = no). Their judgements would also reverse in the *power* and *nice tasks*. This would be reflected by an effect of the interaction of task and children's gender. Moreover, there would be an age effect on children's judgements in which older children would be more likely to show gender stereotypes. Together, the above hypotheses would be reflected by the results in which children's judgements will be influenced by factors related to their age, gender and task.

## 2. Dyadic context tasks

For both dyadic tasks and collective tasks, we preregistered our hypotheses, method, primary analyses and sample size (https://osf.io/rjt6h/?view_only=d33c09eefd1142da9b18aad6d45996fd).

### 2.1. Method

#### 2.1.1. Participants

We determined our sample size based on the methods described by Charafeddine *et al*. [48, experiment 1a]. We recruited a comparable sample size of 60 (30 girls and 30 boys) for each age group: 4-, 5-, 6- and 7-year-olds. The total sample size was 240 children. Their caregivers were recruited via a major Japanese Internet survey company (Cross Marketing Inc., Tokyo, Japan). The parents were randomly recruited from Japan (e.g. 11.3% from Tokyo and 6.5% from Osaka). Twenty-eight parents (11.6%) were male, and other parents (88.4%) were female. Socio-economic status was assessed by parental education. Parental education level was assigned a value from 1 to 5 as follows: 1, less than high school (0%/ 0.5%, male/female parents); 2, high school (14.3%/13.1%); 3, some college (14.3%/30.6%); 4, undergraduate degree (50%/51.3%) and 5, graduate level (21.4%/4.5%). Data collection was stopped after achieving the planned sample size. There were no significant differences in age (in months) between the girls and boys (*p* = 0.339). The children did not have any known developmental disabilities. Informed consent (included in the online survey) was obtained from all parents before their child's involvement in the study, which was conducted following the principles of the Declaration of Helsinki and approved by the local ethics committee.

#### 2.1.2. Procedure

**Figure 1.**
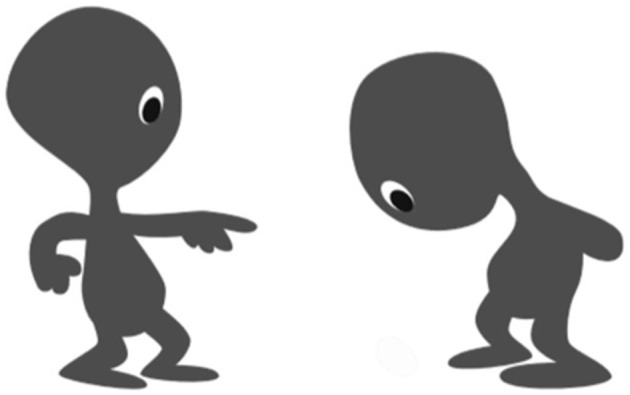
Stimuli used in the dyadic *power task*. Two characters display dominant/subordinate postures [48].

The participants performed the task with their parents. The caregivers controlled the online survey, explained the tasks according to the presented instructions and entered the children's answers. There were two tasks in the dyadic context tasks. The *power task* was inspired by Charafeddine *et al*. with some modifications [48]. In this task, children were given a drawing of two non-gendered characters interacting with each other; one displays a dominant posture, and the other displays a subordination posture (figure 1). The children were told that the former character was saying: ‘You have to do everything I say! Do what I want!’ and the latter character was replying: ‘Ok! I will do what you want’. Two questions were then asked: one concept question, where children were asked to judge which character was more powerful (in Japanese, *Dochira ga erai?*), and one gender stereotype question, in which the children were told that the picture depicted a girl and a boy and were asked to indicate who was who.

**Figure 2.**
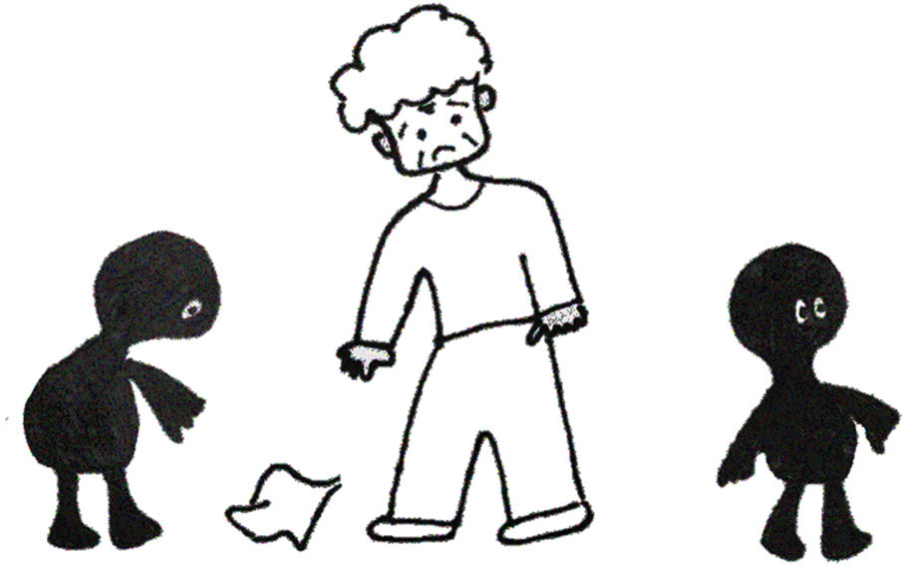
Stimuli of the characters used in the dyadic *nice task*. The elderly person has dropped his handkerchief. One character is going to pick up the handkerchief, while the other seems uninterested in helping.

In the second *nice task*, the children were given a drawing of an elderly person in trouble; one character helped the elderly person, while the other did not (figure 2). As with the first task, a concept question asked children to judge which character was nice (in Japanese, *Dochira ga yasashii?*). As mentioned above, they were told that the picture depicted a girl and a boy and were asked to indicate who was who.

#### 2.1.3. Statistical analyses

The analysis followed Bian *et al*. [45] and Okanda *et al*. [60], using own-gender-judgement scores (scored as 1 if children chose their gender, otherwise 0) regarding the ‘nice’ and ‘powerful’ questions. Children were excluded from the final analysis if they did not answer the concept questions correctly. We conducted a generalized linear mixed model (GLMM) with binomial error distribution and logit link function to determine whether the child identified the dominant/nice character as their own gender. Fixed effects included task (power versus nice), age (comparing 4-, 5-, 6- and 7-year-olds), gender (boys versus girls), and all possible interaction terms. We also added parents' gender (fathers versus mothers) to the fixed effects as a controlling factor. The children's identities (i.e. ID numbers) were included as a random intercept. We simplified the model by applying the backward elimination of non-significant terms. The likelihood-ratio test (LRT) was used in the elimination step. When the final model included an interaction term, we conducted follow-up pairwise comparisons, and *p*-values were adjusted using Holm's (1979) method.

All analyses were conducted in R 4.0.3 [69]. We used the lme4 package [70] for model fitting and the buildmer package [71] for backward elimination. We used the emmeans package [72] for *post hoc* comparisons.

### 2.2. Results and discussion

To ensure that the final sample did not include children who could not correctly comprehend the context (e.g. the relationship between the characters) in the *power task* and *nice task*, we excluded children from the final analysis if they did not correctly answer both concept questions. This led to an exclusion of 65 children (27.1%). The following analyses were performed on the remaining 175 children. There was no difference in the proportion of participants excluded from analysis by age, LRT: $\chi_{3}^{2}=4.806,p=0.186$, or gender, LRT: $\chi_{1}^{2}=3.658,p=0.056$. Moreover, the passing rate of the concept questions in the *power task* in the current study (79.2%) did not deviate from that of the study by Charafeddine *et al*. [48], in which 76.8%–89.3% (95% CI) of children correctly matched the erect body posture with the power statement.

**Figure 3.**
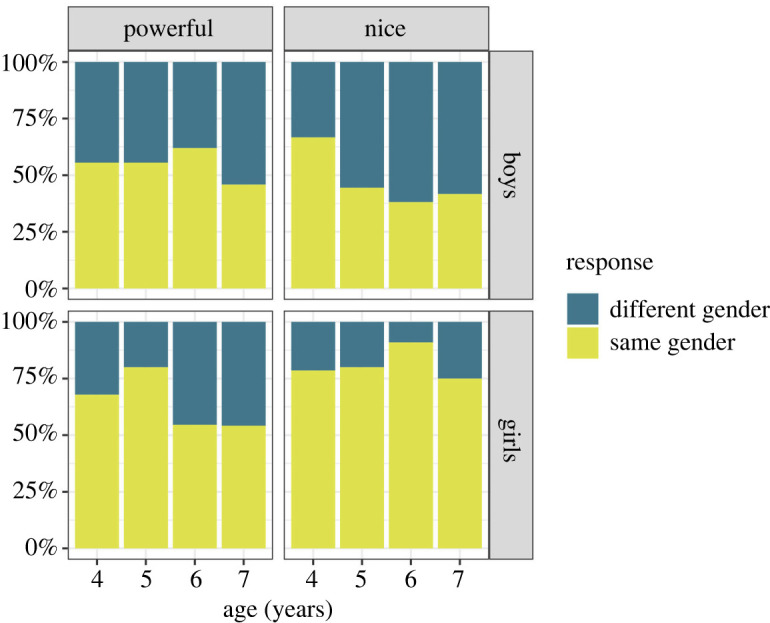
Proportion of children's responses in the dyadic context tasks.

Figure 3 shows the proportion of children's gender judgements for each task by gender and age. The GLMM showed that children's judgement was not affected significantly by age or any of the interaction terms including age, LRT: $\mathrm{Age}\times \mathrm{Gender}\times \mathrm{Task}:\;\chi_{3}^{2}=5.115,p=0.164;\;\mathrm{Gender}\times \mathrm{Age}:\;\chi_{3}^{2}=1.271,$ $p=0.736;\mathrm{Age}\times \mathrm{Task}:\;\chi_{3}^{2}=0.964,p=0.810;\mathrm{Age}:\;\chi_{3}^{2}=2.818,p=0.420$. The final model included the main effect of task, LRT: $\chi_{1}^{2}=1.285,p=0.257$, the main effect of gender, LRT: $\chi_{1}^{2}=17.476,p<0.001$, and the significant interaction between task and gender (LRT: $\chi_{1}^{2}=6.518,p=0.011$) (see also electronic supplementary material, table S1). The effect of parents' gender was not significant, LRT: $\chi_{1}^{2}=1.097,p=0.295$. The follow-up pairwise comparison revealed that girls were significantly more likely than boys to identify the nice character as their own gender (*p* < 0.001), but there was no difference between boys and girls in their tendency to identify powerful characters as their own gender (*p* = 0.208). Furthermore, girls were more likely to identify their own gender as the nice character than as the powerful character (*p* < 0.020), but there was no difference between the *power task* and *nice task* in boys’ tendency to identify their own gender character (*p* = 0.345).

To explore the factors driving the main effects of task and gender, we applied exploratory analyses to test whether children's judgements differed from chance. The analyses were conducted by task and gender. We conducted intercept-only generalized linear models (GLMs) with Bernoulli error distribution and a logit link function to test whether the child predicted the dominant/nice character as their own gender (1 = yes, 0 = no). Holm's *p*-value adjustment was used for multiple comparisons. The results showed that girls identified both the nice and powerful characters as female more frequently than chance (*nice task*: p<0.001; *power task*: p<0.001), but biased judgements were not observed for the boys (*nice task*: p=0.579; *power task*: p=0.437).

Although the results regarding ‘nice’ did not fully support our hypothesis that children (both boys and girls) would be more likely to identify the nice character as female in the nice task, they suggest a ‘nice = female’ gender stereotype. This stereotype indicates that girls exhibit in-group favouritism and/or endorse the ‘nice = female’ stereotype, whereas boys' judgements are neutralized by their in-group favouritism and the ‘nice = female’ stereotype [46–48]. Therefore, the results are consistent with the findings of Okanda *et al*. [60] and suggest that the current experimental procedure and participant sample did not deviate from past investigations [60].

However, regarding ‘power', children's judgements reversed. This suggests a ‘powerful = female’ gender stereotype in children, contrasting past findings in Western children (a detailed discussion can be found in the General discussion). The results did not support our hypothesis that children would be more likely to identify the dominant character as male in the *power task*. In addition, the non-significant effect of age on children's judgements did not support our hypothesis that gender stereotypes would increase by age.

In addition, the exploratory analysis included children who failed in the concept questions and thus were excluded from the final sample. The pattern of the findings did not change whether or not we excluded this subsample (electronic supplementary material, figure S1 and table S2).

In the collective context tasks, children were presented with stories that included verbal cues describing explicitly the powerful and kind characteristics of the protagonists. Moreover, instead of judging whether the characters in dyadic interactions were male or female, children were asked to identify the gender of the protagonists introduced in the contexts, which included multiple unspecified people [45,60].

## 3. Collective context tasks

### 3.1. Method

#### 3.1.1. Participants

The participants were the same as in the dyadic context tasks.

#### 3.1.2. Procedure

The procedure of the collective context tasks was designed based on the study of Okanda *et al*. [60], which tested children's gender stereotypes about ‘nice’ and ‘brilliant’. Okanda *et al*. [60] included screening questions about the concepts of ‘nice’ and ‘smart’. Similarly, we used the dyadic context tasks' concept questions to check whether children understood the concepts of ‘powerful’ and ‘nice’.

**Figure 4.**
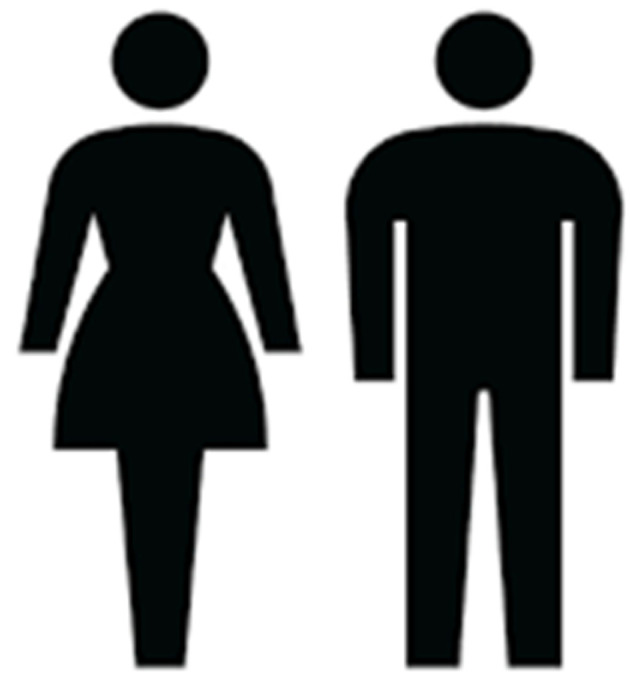
The stick-figure stimuli used in the collective context tasks.

In the collective context tasks, children were asked to choose stick humans (drawn in black; figure 4) to indicate a person's gender; such stick figures can avoid the effect of perceptual cues [60]. Before the main tasks, children were asked to identify the gender of each stick figure (e.g. ‘Which figure depicts a woman?’).

The collective context tasks comprised two main tasks. Task (i) consisted of two stories describing unfamiliar adults whose gender was purposely unspecified. The stories were designed based on the study by Bian *et al*. [45]. In the *power story*, children were told, ‘There are lots of people at the place where I work. But there is one person who is really special. This person is really, really powerful [*erai*]. This person is in an especially strong position and can give orders to other members. This person is really, really powerful.’ Previous studies have shown that at approximately 4 years old—the age of the youngest participants group of the current study—children map decision-making power (i.e. giving permission, setting norms) onto social status (e.g. ‘in charge', ‘the boss’; [73,74]).

In the *nice story*, children were told, ‘There are lots of people at the place where I work. But there is one person who is really special. This person is really, really nice [*yasashii*]. This person likes to help others with their problems and is friendly to everyone at the office. This person is really, really nice.’ After each story, the children were shown four stick figures in a line (two female/two male) and asked to guess which one of the four people might be the person in the story. In task (ii), children were shown four pictures individually; each picture depicted two stick figures indicating a male and a female. The children were told that one of the two people had power (on two of four trials) or was nice (on the other two trials), and they were asked to guess which of the two stick figures had the relevant trait. The order in which the pictures were shown was pseudorandom.

#### 3.1.3. Statistical analyses

The coding and analysis procedures were identical to those used in the dyadic context tasks. We checked whether the children passed the concept of *power* and *nice tasks* in the dyadic context tasks. In addition, we checked whether children answered the question related to the gender of the stick figures correctly. The final analysis used in the collective context tasks included only children who answered these questions correctly. To examine the factors influencing children's gender judgement in each task, we conducted a GLMM with binomial error distribution and logit link function for children's own-gender judgement scores. Fixed effects included task (power versus nice), age (comparing 4-, 5-, 6- and 7-year-olds), gender (boys versus girls), and all possible interaction terms. We also added parents' gender (fathers versus mothers) to the fixed effects as a controlling factor. Children's identities (i.e. ID numbers) were included as a random intercept. Statistical treatments, such as model simplification, LRT and *p*-value adjustment, were identical to those used in the dyadic context tasks.

### 3.2. Results and discussion

After excluding children who did not answer the conceptual and gender questions correctly (70, 29.2%), we analysed the data from the remaining 170 children. There was no difference in the proportion of participants excluded from analysis by age, LRT: $\chi_{3}^{2}=6.593,p=0.086$, or gender, LRT: $\chi_{1}^{2}=2.079,p=0.149$.

**Figure 5.**
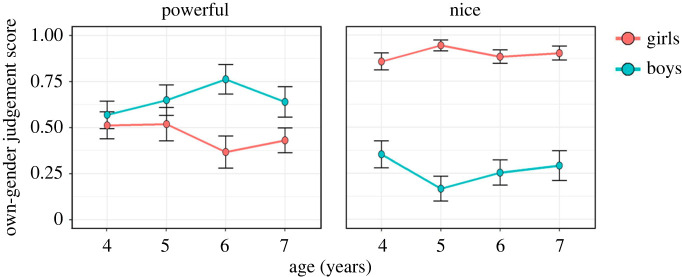
Children's own-gender judgement scores by task, gender and age. Dots and error bars represent means and ± 1 s.e., respectively.

The results of GLMM revealed a significant main effect of gender, LRT: $\chi_{1}^{2}=7.016,p=0.008$, a significant interaction between task and gender, LRT: $\chi_{1}^{2}=148.115,p<0.001$, and a significant interaction among task, gender and age, LRT: $\chi_{3}^{2}=10.033,p=0.018$. The final model included all possible interaction terms (figure 5; see also electronic supplementary material table S3). The effect of parents' gender was not significant, LRT: $\chi_{1}^{2}=0.108,p=0.742$.

Since the final model showed that the task effect depended on gender and age, follow-up pairwise comparisons were performed to compare mean own-gender judgement scores between the *power* and *nice tasks* within each gender and age. We found that in all age groups, the boys showed higher scores in the *power task* than in the *nice task* (4-year-olds: p=0.020; 5-year-olds: p<0.001; 6-year-olds: p<0.001; 7-year-olds: p<0.001). In contrast, the girls scored higher in the *nice task* than the *power task* for all age groups (ps<0.001).

Considering the final model showed that the gender effect depended on task and age, follow-up pairwise comparisons were performed to compare mean own-gender judgement scores between the boys and girls within each task and age. In the *power task*, only 6-year-old boys showed significantly higher scores than girls (6-year-olds: p<0.001), but there were no significant differences by participant gender in the other age groups (4-year-olds: p=0.591; 5-year-olds: p=0.463; 7-year-olds: p=0.086). In the *nice task*, girls scored significantly higher than boys in all age groups (ps<0.001).

The results did not reveal a consistently positive effect of age on children's judgements regarding the gender stereotype of social power, which did not support our hypothesis (see the General discussion for possible explanations).

We compared the own-gender judgement scores with the chance level for each task, age and gender as an exploratory analysis. We conducted a GLM with binomial error distribution and a logit link function for own-gender judgement scores. We set an intercept-only model to test the difference from chance level in each task, age and gender with Holm's *p*-value adjustment. In the *power task*, 6-year-old boys showed significantly higher own-gender judgement scores than the chance level (6-year-old boys: p<0.001), but the scores of the other boys' age groups and girls were not significantly different from the chance level (4-year-old boys: p=0.986; 5-year-old boys: p=0.224; 7-year-old boys: p=0.160; 4-year-old girls: p=1.000; 5-year-old girls: p=1.000; 6-year-old girls: p=0.231; 7-year-old girls: p=0.960). In the *nice task*, girls scored significantly higher than chance levels in all age groups (ps<0.001). In the nice task, the scores of 4-year-old boys were not significantly different from chance level (4-year-old boys: p=0.231), whereas the scores of 5–7-year-old boys were significantly lower than chance levels (5-year-old boys: p<0.001; 6-year-old boys: p=0.002; 7-year-old boys: p=0.006). The findings revealed that children have gender stereotypes about ‘nice’. Even for the youngest group (i.e. 4-year-olds), the girls tended to attribute ‘nice’ to females. Importantly, the ‘nice = female’ belief can also be observed in boys, although it requires more development time (i.e. from 5 years of age). These findings were consistent with the findings of the dyadic context tasks and the findings of a previous study conducted with Japanese children [60].

Children's judgements contrasted between ‘power’ and ‘nice’. Boys were more likely to attribute ‘powerful’ than ‘nice’ to males, and girls were more likely to attribute ‘nice’ than ‘powerful’ to females. These findings show that rather than indiscriminately attributing particular properties to their gender, children make judgements based on their beliefs regarding different domains of social evaluation.

The results partially supported our hypothesis, which posited that children would be more likely to identify the nice character as female in the *nice task*, whereas children would be more likely to identify the dominant character as male in the *power task*. Specifically, although children's judgements varied by task, the patterns of their gender-biased judgements in the tasks depended on the children's gender. Girls did not show biased gender judgements about social power in any age group; boys selectively attributed the property of ‘powerful’ to males at 6 years of age (7-year-old boys showed a consistent but weaker bias).

Exploratory analysis included children who failed in the conceptual and gender questions and thus were excluded from the final sample. The pattern of the findings did not change whether or not we excluded this subsample (electronic supplementary material, figure S2 and table S4).

## 4. General discussion

The belief that males are more powerful than females leads to undesirable gender disparities, but little is known about the developmental origins of this belief, especially in Eastern cultures. The current study investigated whether and from what age Japanese children held ‘powerful = male’ beliefs regarding dyadic interaction context and collective occupational context, respectively. Moreover, to test the specificity of the gender stereotype of social power, we considered whether children's beliefs depend on personal traits. Specifically, we compared children's gender stereotypes about ‘powerful’ and ‘nice’.

In the dyadic context tasks, we tested whether children are more likely to attribute power to boys than girls in boy–girl interactions, which has been found in several cultures [48]. Children saw pairs of drawn characters depicting powerful–powerless or kind–unkind postures and judged the gender of the powerful/nice character. As a new approach, we introduced the gender judgement task regarding the personal trait of ‘nice’ as a point of comparison [45,60]. The results indicated a gender stereotype of ‘nice = female', whereas the ‘powerful = male’ gender stereotype was not observed. Specifically for the *nice task* girls (but not boys) associated ‘nice’ with females. This demonstrates that the ‘nice = female’ stereotype may be endorsed by children as young as 4 years, showing consistency with the past findings on Japanese children [60]. Boys did not show biased judgements for the power task, while girls were likelier to associate power with girls than boys, suggesting a possible ‘powerful = female’ belief.

The findings that female participants associated their gender with both ‘nice’ and ‘powerful’ might be interpreted as girls' own gender preference [43], which has been suggested by past findings [48,67,68]. However, past studies have revealed that children significantly associate powerful postures with boys, drawing differences from the current findings [48]. Assuming that the power concept (including the word *erai*) is understood as a positive, non-stereotyped trait, one would expect that girls and boys would significantly associate it with their gender. However, past studies have shown that when presented with the same stimulus used in the current study, Japanese children exhibit a less positive attitude (i.e. at chance) towards dominance compared with French children. This suggests that unlike children with Western cultural backgrounds, Japanese children may not demonstrate in-group favouritism regarding dominance-gender matching tasks [49,50]. Consequently, the current findings probably reflect children's gender stereotypes regarding power rather than in-group favouritism or the interaction of stereotype and in-group favouritism.

Therefore, it appears that the stimulus evokes feminine stereotypes rather than masculine or neutral ones. Regarding this interpretation, we would like to note that it does not seem that the word *erai*, which was not used in past studies but in the current study, influenced children's judgements. Because the passing rate of the concept questions in the power task in the current study was comparable to that of the past study, and the final sample included only the children who correctly answered the concept questions, then it would be appropriate to conclude that the majority of the participants have correctly matched the erect body posture with the statement of *erai*. Moreover, after including the participants who failed in the concept questions, the findings did not change much—girls, but not boys, associated both ‘nice’ and ‘power’ with their genders (see electronic supplementary material, Information). This suggested that children made gender judgements mainly based on information about the visually postural relationships between the characters rather than their understanding of the meaning of the word *erai*. Nevertheless, because the concept of *erai* has positive connotations, if children made judgements while considering this positivity, then the findings may reflect not only gender stereotypes but also in-group favouritism. However, this explanation does not conflict with the explanation of the ‘powerful = female’ stereotype (because if this stereotype did not exist, boys would have shown ‘powerful = male’ judgements).

Furthermore, we propose two accounts that could form the ‘powerful = female’ association identified in the dyadic girls–boys context. First, it may result from the participants' everyday peer interactions. Empirical evidence has demonstrated that in Japanese kindergartens, boys are more prone than girls to engage in norm-violated play activities (e.g. speaking taboo words related to excretion and sex, teasing other children [75]). Thus, compared with girls, boys would receive warnings more frequently from both teachers and peers. In the dyadic context of girls and boys, the subordinate character's posture signifies a ‘being warned’ status. This would have led the participating children to associate the subordinate character with boys and, consequently, the dominant character with girls. This account requires further testing since it does not explain why children in other cultures associate an erect body posture with boys. This is especially notable given that the phenomenon of boys engaging in norm-violated play activities appears to be culture-independent [76]. It would be valuable to examine how children in various cultures qualitatively assess the relationships between characters in the dyadic girls–boys context, including their spontaneous interpretation of the stimuli context.

The second account is based on the reliance on Japanese cultural norms that participants experience within their families daily. This account would bear more significant implications for the early development of stereotypes about social power, suggesting that the gender stereotype ‘powerful = male’ develops based on environmental information people receive. In Japanese culture and society, the expression ‘*kakaa denka* (a family whose wife holds a stronger position than her husband)’ has existed for a long time [77]. This term refers to a situation in which wives hold substantial authority and influence within the household, expressing the wife's leading role, particularly in economic matters and child-rearing. In traditional Japanese family structures, while spouses commonly cooperate, it is typical for the wife to take the lead in managing the household (note that this represents a general tendency, and individual households and circumstances may exhibit variations). Therefore, many children might have learned that in personal situations such as dyadic interactions, females are more likely than males to play a dominant role.

The methodological difference between the current study and past studies should be noted. In Charafeddine *et al*. [48], there was no use of a different concept (i.e. ‘*erai*’) than the dialogue presented at first [48]. Then, children were asked to map each utterance to one of the characters in a counterbalanced order and then to infer the gender of each character. In contrast, in the present study, children were only asked to designate the powerful character in the conceptual question, which may make this character more salient and does not lead the children to make the association from gender to submissiveness. Past studies have suggested that the stereotype of ‘submissive = female’ may be operant as the stereotype of ‘dominant = male’ [46]. Although we do not think that this methodological difference could explain the observed cultural difference in children's judgements, it would be interesting to examine whether and how children's responses vary when they are asked to focus on the dominant role, the submissive role, or both roles.

In the collective context tasks, children were presented with stories that included verbal cues that explicitly described the personal traits of the protagonists. Moreover, instead of judging whether the characters were male or female in a dyadic interaction, children were asked to identify the gender of a socially powerful/nice adult in the context, including unspecified people [45,60]. Similar to the findings of dyadic context tasks, we found that girls and boys were likelier to associate ‘nice’ with females. However, girls did not show biased gender judgements regarding power, whereas, from the age of 6, boys seem more likely to associate power with their gender than girls. This suggests that signs of the ‘powerful = male’ gender stereotype emerge from about six years of age.

The findings regarding the power gender stereotype are consistent with the findings in American children, which showed that from 6 years old, boys (but not girls) attribute power to same-gendered adults in occupational contexts (despite that Japanese children seem to hold this belief more weakly than American children do [51, experiment 1]).

In Japanese culture and society, most top positions in the economic and political fields have been held by men [78]. This state of affairs could be absorbed by children in various ways, such as through television and the Internet. Thus, when the children were asked to judge whether a man or a woman was the most powerful person in the workplace, they would recall the ‘powerful = male’ information and make a biased judgement.

Children's beliefs regarding power in dyadic and collective context tasks showed different tendencies. As mentioned above, we propose this is due to social norms in Japanese culture. Japanese culture shows a divergence between power dynamics within the family and gender roles or power relations in public settings: women may play more leading roles within the family, while gender power dynamics are reversed in public settings. Compared with the former, power dynamics in the latter context might reflect a greater variance of power within males than within females. That is, a ‘powerful = male’ hierarchical structure is expected to be formed by males occupying the very top (and bottom) of the dominance hierarchies due to the greater selective pressure for intrasexual competition faced by males than by females [79,80]. As a result, a general ‘powerful = male’ stereotype might be maintained by an over-representation of males in powerful positions in collective contexts (e.g. group context), which includes greater competition pressure.

Even though collective context tasks revealed signs of children's gender stereotypes about social power, it seems that Japanese children take longer than children in other cultures (e.g. American) to develop such gender stereotypes [48,51]. The late development of gender stereotypes in Japanese children has been observed in other domains of personal traits. For instance, Bian *et al*. [45] showed that from 6 years old, American girls become less likely than American boys to believe that members of their gender are ‘really, really smart’. Okanda *et al*. [60] implemented similar tests in Japanese children, showing that the ‘brilliance = male’ belief does not emerge until age 7. Given the possibility of a potential link between the beliefs about brilliance and social power (e.g. they may be confounded with the trait of competence), it might not be surprising that Japanese children develop gender stereotypes of power and brilliance at a similar age. Together, the findings suggest not only that gender stereotypes are partially learned traits but also that the developmental processes of gender stereotypes may depend on culture.

We hypothesized that age positively influences children's gender stereotypes regarding social power because it was easy to imagine that the accumulation of experience as children learn information about gender differences from their environment would strengthen their biased beliefs [45,60,63–68]. However, the current study did not reveal a consistently positive effect of age. The lack of a consistently positive effect of age has also been observed in the previous study using similar tasks [48]. One reason might be the limited age range of the children. Previous studies that revealed age's influence on gender stereotypes regarding social power included school-age children, adolescents and adults—participants older than the current sample [67,68,81]. Thus, it might be that the information young children receive to strengthen their gender stereotypes is still too little or too homogeneous across ages to elicit the influence of age on the stereotype. Another reason might be that because children's expectation of equality in individual interactions increases with age [82,83], the possible positive influence of age on the stereotype might be neutralized by developing egalitarianism. Of course, the assumption regarding the positive influence of gender stereotypes of power itself might need further evaluation to test whether it is appropriate to use social learning theories to explain the development of gender stereotypes. Further research is needed to answer these questions.

The current tasks consistently revealed children's gender stereotype of ‘nice = female’ in both dyadic and collective contexts. Croft *et al*. [84] proposed the issue that even though past studies had sought to understand and mitigate the psychological barriers that block women from entering previously male-dominated activities, less corresponding research has examined men's under-representation in communal roles traditionally occupied by women (e.g. careers in healthcare, early childhood education, and domestic roles including child care). The current findings demonstrated that the biased beliefs that females are nicer (kinder) than males can be observed in 4-year-old children, suggesting a very early acquisition of the stereotype. Because holding such stereotypes may constrain boys' interest in acting as nice persons and impair their performance when working previously female-dominated activities, future empirical investigations and practical interventions are needed to look deeper into the stereotype and make efforts to reduce the biased beliefs.

It is worth discussing several research directions for future studies. First, Neff *et al*. [81] examined perceptions of inequality (e.g. ‘Who tends to have more power to make decisions in [politics/the business world/the home], men or women?’) among early, middle and late adolescents. They found that men were seen to have more power and status in politics than in business, whereas relative equality was seen to exist in the home. Considering the current findings that children attribute power to ‘men’ but not ‘boys’, it would be interesting to test whether and how children's stereotypes vary toward people with different demographic characteristics (e.g. occupation, generation).

Second, gender stereotypes hinder women's success in many fields (e.g. science, leadership) from childhood [85–87]. Recently, psychologists have found that subtle linguistic cues can increase girls' engagement in science [88]. Specifically, language describing science as action (Let's do science! Doing science means exploring the world!) rather than in terms of identities (Let's be scientists! Scientists explore the world!) led to more persistence among girls in science games designed to teach children about the scientific method. This suggests that removing language marking identity labels can avoid the negative effect of gender stereotypes on girls' engagement in scientific activity. Regarding the gender stereotype of power, it is worth investigating whether children's interest in being a leader depends on how the position is presented to them. For instance, future research may test how interested children are in becoming a ‘president’, ‘politician’, or a ‘leader of a company’. This would be followed by testing whether children's interests change if they were asked to make rules to make a better country or company.

Related to this point, it is worth noting that the concept of ‘*erai*’ may encompass both perspectives of leadership and power. Past studies have found that when high status is not framed in terms of power but in terms of leadership, Western children do not necessarily link it to gender [89]. Moreover, when asking adults about children's gendered traits [90,91], ‘leader’ does not emerge as a stereotype of boys, whereas ‘dominant’ (e.g. aggressive) does. Future studies should investigate how children's gender stereotypes vary across different types of high-ranked positions.

There were some marginally significant results regarding data exclusion. Although the non-significant *p*-values suggest that the effects are incidental, it is valuable to speculate as to why this might be and what follow-up research might be encouraged. Specifically, concerning the dyadic context tasks, the number of children who could not correctly comprehend the relationship between the characters in the *power task* and the *nice task* seems to vary by gender (with 39 boys and 26 girls excluded). Moreover, in the collective context tasks, the number of children who did not correctly answer the conceptual and gender questions seemed to vary by age (with 15 4-year-olds, 24 5-year-olds, 19 6-year-olds, and 12 7-year-olds excluded). Such potential variations were not observed in Okanda *et al*. [60], which was conducted in Japan using similar experimental methodologies [60]. More importantly, the current findings did not change whether these children were excluded from the analysis or not. The potential variations seem to lack theoretical rationales. For instance, although there may be some variation depending on age, it does not seem like the youngest or oldest children are disproportionately excluded (e.g. a linear pattern). However, because the current study was conducted under caregiver–child interaction in family settings, there may be factors that influence children's performance at specific ages or with specific genders (e.g. schedules and amounts of lessons and classes may vary depending on age or gender). Future research is expected to pay more attention to these possibilities, contributing to promoting the validity of investigations.

Two limitations of the current study should be noted. The first limitation is the potential influence of caregivers. We did not have visual access to the participants to check whether they were paying attention to the tasks, so it is possible that the children's performance was influenced by the caregivers, who we expect to possess internalized gender stereotypes regarding social power considering the large gender inequality in social power in Japan [11,12]. This possibility does not seem to be supported by the current findings, particularly because if caregivers influenced children's judgement, we would expect stronger gender stereotypes in younger children (e.g. 4-year-olds) who would probably have required more explanation for completing the task. In addition, the caregiver's gender may have influenced participants' judgements; for example, a female authority might have made the notion of female power more salient and increased the tendency of children to attribute power to the female character. This might have contributed to the current findings, which showed a lack of evidence for the ‘powerful = male’ stereotype in most cases. However, this possibility is not supported by the findings, given that children's judgements were not influenced by whether they participated in the study with their mother or father.

It is not surprising that the large majority of the parents who interviewed their children are females. First, this mirrors a gender asymmetry that is common in online surveys. Second, mothers are more frequently the primary caregivers in families even today. This family context may have influenced the perception of the ‘*erai*’ individual as more feminine in a dyadic context (e.g. mother–father interaction; similar to the second account for the ‘powerful = female’ gender stereotype mentioned above) than in a collective context. Future studies are expected to investigate whether children show similar responses in other settings, such as in school settings wherein significance of the situation is more about inter-children relations. Or, applying different tasks such as that the characters children compare were randomly selected from a group with other members.

Moreover, if caregivers influenced children's judgement, we would expect a higher passing rate on the comprehension tests in younger children, given that younger children would be more dependent on caregivers for completing tasks [48]. However, this was not observed: in the current study, children's passing rates on comprehension tests did not vary by age. Although we acknowledge that the caregivers might have influenced the children's performance, the findings show that this risk is low. However, laboratory experiment replications would be useful to extend and generalize the current findings.

The second limitation is that although children's performance on the concept question of power did not deviate from that of Charafeddine *et al*. [48], it is unclear whether children comprehend power as a personal trait, like niceness or situational behaviour. It is also unclear whether children of different ages view power as good or bad, which might affect their responses, especially among older children with greater concern for social approval. Future investigations are needed to understand children's social evaluation regarding power better.

In summary, the current study provides evidence that Japanese children's beliefs regarding gender differences in power vary depending on the context in which male–female interactions are presented. Incorporating findings from Western cultures, the current study highlights the significant influence of cultural information on the development of gender stereotypes.

## Data Availability

The data are provided in electronic supplementary material [92].
